# Characterization of three predicted zinc exporters in *Brucella ovis* identifies ZntR-ZntA as a powerful zinc and cadmium efflux system not required for virulence and unveils pathogenic Brucellae heterogeneity in zinc homeostasis

**DOI:** 10.3389/fvets.2023.1323500

**Published:** 2024-01-08

**Authors:** Beatriz Tartilán-Choya, Carmen Tejedor, Raquel Conde-Álvarez, Pilar María Muñoz, Nieves Vizcaíno

**Affiliations:** ^1^Departamento de Microbiología y Genética, Universidad de Salamanca, Salamanca, Spain; ^2^Instituto de Investigación Sanitaria de Navarra and Departamento de Microbiología y Parasitología, Universidad de Navarra, Pamplona, Spain; ^3^Departamento de Ciencia Animal, Centro de Investigación y Tecnología Agroalimentaria de Aragón (CITA), Zaragoza, Spain; ^4^Instituto Agroalimentario de Aragón-IA2 (CITA-Universidad de Zaragoza), Zaragoza, Spain; ^5^Instituto de Investigación Biomédica de Salamanca (IBSAL), Salamanca, Spain

**Keywords:** Brucella ovis, zinc, cadmium, cobalt, ZntA, ZntR, ZnuA, virulence

## Abstract

*Brucella ovis* causes non-zoonotic ovine brucellosis of worldwide distribution and is responsible for important economic losses mainly derived from male genital lesions and reproductive fails. Studies about the virulence mechanisms of this rough species (lacking lipopolysaccharide O-chains) are underrepresented when compared to the main zoonotic *Brucella* species that are smooth (with O-chains). Zinc intoxication constitutes a defense mechanism of the host against bacterial pathogens, which have developed efflux systems to counterbalance toxicity. In this study, we have characterized three potential *B. ovis* zinc exporters, including the ZntA ortholog previously studied in *B. abortus*. Despite an in-frame deletion removing 100 amino acids from *B. ovis* ZntA, the protein retained strong zinc efflux properties. Only indirect evidence suggested a higher exporter activity for *B. abortus* ZntA, which, together with differences in ZntR-mediated regulation of *zntA* expression between *B. ovis* and *B. abortus*, could contribute to explaining why the Δ*zntR* mutant of *B. abortus* is attenuated while that of *B. ovis* is virulent. Additionally, *B. ovis* ZntA was revealed as a powerful cadmium exporter contributing to cobalt, copper, and nickel detoxification, properties not previously described for the *B. abortus* ortholog. Deletion mutants for BOV_0501 and BOV_A1100, also identified as potential zinc exporters and pseudogenes in *B. abortus*, behaved as the *B. ovis* parental strain in all tests performed. However, their overexpression in the Δ*zntA* mutant allowed the detection of discrete zinc and cobalt efflux activity for BOV_0501 and BOV_A1100, respectively. Nevertheless, considering their low expression levels and the stronger activity of ZntA as a zinc and cobalt exporter, the biological role of BOV_0501 and BOV_A1100 is questionable. Results presented in this study evidence heterogeneity among pathogenic Brucellae regarding zinc export and, considering the virulence of *B. ovis* Δ*zntA*, suggest that host-mediated zinc intoxication is not a relevant mechanism to control *B. ovis* infection.

## 1 Introduction

*Brucella ovis* is a gram-negative bacterium of the genus *Brucella* that causes a serious ovine reproductive disease manifested by male genital lesions, reduced fertility, abortion, and consequent economic losses (1, 2). Most *Brucella* spp. are smooth bacteria exposing an O-polysaccharide (O-PS) linked to the lipid A-core oligosaccharide. This is the case of *B. melitensis, B. abortus,* or *B. suis,* which preferentially infect sheep/goats, cattle, and pigs, respectively, and cause notifiable reproductive and zoonotic diseases. In smooth brucellae, the O-PS is critical for virulence and carries the main epitopes involved in their serodiagnosis (3–5). By contrast, *B. ovis* is a non-zoonotic rough bacterium with a lipopolysaccharide devoid of O-PS, which also plays an essential role in its virulence and immunogenicity (6).

The unique vaccine available against sheep and goat brucellosis is the live-attenuated *B. melitensis* Rev. 1 smooth strain, which confers protection against both *B. melitensis* and *B. ovis* infection (1, 7). However, since Rev. 1 is infective for humans and induces antibodies against the O-PS, which can interfere with the routine diagnosis required for *B. melitensis* surveillance, this vaccine is banned in *B. melitensis-*free areas. Consequently, the incidence of *B. ovis* infection is increasing in some of these regions (2, 8), which calls for the development of a *B. ovis-*specific vaccine. Ideally, this vaccine should be safe (non-zoonotic) and not trigger O-PS antibodies. In this context, obtaining attenuated *B. ovis* strains with potential use as *B. ovis* homologous vaccine is of interest. Although studies regarding *B. ovis* are progressively increasing in number, most knowledge about the mechanism and genes involved in *Brucella* virulence has been obtained from the smooth *B. melitensis* and *B. abortus* species. Further research on *B. ovis* virulence factors and host–pathogen interaction mechanisms is needed to acquire proper knowledge toward the development of vaccine candidates.

An inherent trait of pathogenic smooth and rough Brucellae is that they are facultative intracellular bacteria able to build a replicative niche inside phagocytes that provides a “safe” environment, favoring their escape from the host immune response and the establishment of chronic infections. Accordingly, *Brucella* mutants showing defects in intracellular survival are usually attenuated in virulence in the natural host and laboratory animals (4). Although the availability of nutrients in the host is a general requirement for the survival and multiplication of bacterial pathogens, it is even more relevant in the restricted intracellular environment. Metal ions are essential micronutrients for various cellular processes involved in bacterial cell viability and virulence, but high concentrations inside the cell lead to serious detrimental effects (9–11). Metals must also be finely balanced in the host, where, in addition, they play an important role in the control of bacterial infections through both metal intoxication mechanisms and a process known as nutritional immunity that restricts metal availability to the pathogen (11). Through evolution, animal hosts have developed several strategies involving metal ions to control the progress of infection, but simultaneously, bacterial pathogens have evolved to minimize the effect of these host defense strategies (10, 11). Since metals cannot be degraded or synthesized by the cell, homeostasis depends on proper coordination of import and export systems (10).

Zinc primarily acts as an enzyme cofactor in bacteria, where 5–6% of proteins are Zn-binding proteins involved in essential metabolic pathways (12). On the contrary, Zn is a highly competitive divalent cation that, when in excess, can displace other metal cofactors from their binding sites in other essential metalloenzymes. To maintain suitable Zn intracellular levels, bacteria have developed three main strategies: (i) Zn-sensing proteins acting as transcriptional regulators, (ii) Zn importer and exporter systems able to mobilize the metal across cell membranes, and (iii) zinc sparing and allocation under extreme Zn scarcity conditions that either increases the levels of Zn-independent proteins with equivalent functions to those performed by Zn-dependent proteins or ensures that Zn is provided, through intracellular chelators acting as Zn reservoirs, to Zn-dependent proteins whose function cannot be replaced (12, 13).

The P_1B_-type ATPase ZntA and its transcriptional regulator ZntR have been linked to Zn^2+^ export in smooth *B. abortus* 2308, and overexpression of *zntA* was proposed as responsible for the attenuation of the Δ*zntR* mutant of this strain (14). However, no additional reports about the relevance of Zn^2+^ efflux systems in other *Brucella* species have been published until now. This type of study with rough *B. ovis* could not only contribute to the development of potential vaccine candidates but also provide interesting information about the similarities and differences among pathogenic brucellae that are highly homologous at the DNA level but have important differences regarding pathogenicity and host preference.

In this study, in addition to the ZntA-ZntR system, two other proteins potentially involved in zinc export were identified by analyzing the *B. ovis* genome. With the aim of determining their relevance for *in vitro* and *in vivo* survival of rough *B. ovis* PA, a panel of 14 genetically modified strains (including deletion mutants, complemented strains, and strains overexpressing selected genes) was constructed and analyzed for gene expression, *in vitro* survival under excess of zinc and other divalent cations, and for virulence in murine macrophages and mice.

## 2 Materials and methods

### 2.1 Cloning vectors, bacterial strains, and culture conditions

PCR-amplified DNA fragments were cloned into pCR^®^-Blunt vector (Life Technologies, Carlsbad, CA, United States) or into pGEM^®^-T Easy (Promega, Madison, WI, United States). Plasmid pCVD-KanD was used to construct the recombinant plasmids employed to delete the selected genes in *B. ovis* PA, according to the procedure summarized below. This plasmid is unable to replicate in *Brucella* and confers resistance to kanamycin and sensitivity to sucrose (15). *Brucella* wild-type genes used to complement deletion mutants or to overexpress selected genes in several strains derived from *B. ovis* PA were cloned into pBBR1MCS4, which confers resistance to carbenicillin and replicates in several copies in the *Brucella* cytoplasm (16).

*Escherichia coli* JM109 competent cells were used to replicate plasmids derived from pGEM®-T Easy and pBBR1MCS4. *E. coli* NZY5α and *E. coli* CC118 were used for the replication of recombinant plasmids derived from pCR^®^-Blunt and pCVD-KanD, respectively. Luria-Bertani agar or broth, supplemented with 50 μg/mL of carbenicillin or kanamycin when required, was used as a culture medium for *E. coli* strains cultured at 37°C.

*B. ovis* PA was used as a parental strain for the construction of the genetically modified strains obtained in this study (Table 1). *B. ovis* strains were cultured at 37°C under a 5% CO_2_ atmosphere and in a culture medium composed of tryptic soy broth or agar (TSB or TSA; Pronadisa-Laboratorios Conda, Torrejón de Ardoz, Spain) supplemented with 0.3% yeast extract (YE; Pronadisa-Laboratorios Conda, Torrejón de Ardoz, Spain) and 5% horse serum (HS; Gibco-Life Technologies, Grand Island, NY, United States). Kanamycin, carbenicillin (50 μg/mL), or 5% sucrose was added to TSA-YE-HS or TSB-YE-HS when required for the selection or maintenance of the genetically modified strains.

**Table 1:** Main *Brucella ovis* bacterial strains used in this study^a^.

Strain name	Relevant characteristics
*B. ovis* PA *B. ovis* PA-pBBR1MCS4 *B. ovis* PA-pNV*zntA*_PA_com5 *B. ovis* PA- pNV*zntA*_2308_com5 *B. ovis* Δ*zntR* *B. ovis* Δ*zntR*-pBBR1MCS4 *B. ovis* Δ*zntR*-pNV*zntA*_PA_com *B. ovis* Δ*zntR-*pNV*zntA*_2308_com *B. ovis* Δ*zntA* *B. ovis* Δ*zntA-*pNV*zntA*_PA_com *B. ovis* Δ*zntA-*pNV*zntA*_2308_com *B. ovis* Δ*A1100* *B. ovis* Δ*0501* *B. ovis* Δ*zntA-*pNV*0501*com *B. ovis* Δ*zntA-*pNV*A1100*com	Parental strain obtained from BCCN ^b^ Parental strain bearing pBBR1MCS4 Parental strain bearing pNV*zntA*_PA_com5 ^c^ Parental strain bearing pNV*zntA_2308_*com5 ^c^ *zntR* deletion mutant of *B. ovis* PA Δ*zntR* mutant bearing pBBR1MCS4 Δ*zntR* mutant bearing pNV*zntA*_PA_com5 ^c^ Δ*zntR* mutant bearing pNV*zntA*_2308_com5 ^c^ *zntA* deletion mutant of *B. ovis* PA Δ*zntA* mutant complemented with pNV*zntA*_PA_com5 ^c^ Δ*zntA* mutant complemented with pNV*zntA*_2308_com5 ^c^ *BOV_A1100* deletion mutant of *B. ovis* PA *BOV_0501* deletion mutant of *B. ovis* PA Δ*zntA* mutant bearing pNV*0501*com ^c^ Δ*zntA* mutant bearing pNV*A1100*com ^c^

a Intermediate bacterial strains obtained during procedures of mutagenesis are not cited.

b BCCN, Brucella Culture Collection Nouzilly (Institut National de la Recherche Agronomique, Nouzilly, France).

c Multicopy plasmids replicating in *B. ovis* bearing the wild type gene specified in the plasmid name (*zntA, BOV_0501 or BOV_A1100* from *B. ovis* PA or *zntA* from *B. abortus* 2308).

Growth curves in TSB-YE-HS started with bacterial cultures of optical density at 600 nm (OD_600_) values of 0.05 that were incubated for 3 days at 37°C under a 5% CO_2_ atmosphere and agitation at 120 rpm. OD_600_ values were recorded at several time points. The growth curves shown in the figures are representative of three independent experiments with similar results. For evaluation of metal toxicity, 5 mM ZnCl_2_, 0.2 mM CdCl_2_, 0.5 mM CoCl_2_, 1 mM CuCl_2_, 1 mM NiCl_2_, 0.8 mM PbCl_2_, 2 mM MnCl_2_, 2 mM FeCl_2_, or 4 μM HgCl_2_ (Sigma-Aldrich, St. Louis, MO, United States) was added to the culture medium at the beginning of the experiment. The concentrations for each compound were previously selected (data not shown) and correspond to concentrations slightly inhibiting the growth of the parental strain.

### 2.2 *In silico* analysis of DNA and protein sequences, primers, and nucleic acid procedures

The *B. ovis* 63/290 (ATCC 25840) whole genome published sequence (GenBank accession numbers NC_009505 and NC_009504 for chromosomes I and II, respectively) was used to retrieve the nucleotide sequences of the target genes and to design the primers (IDT, Leuven, Belgium) used in this study (Table 2). Genes of *B. abortus* 2308 were also obtained from the published whole genome (GenBank accession numbers AM040264 and AM040265). The genome database of the Kyoto Encyclopedia of Genes and Genomes (KEGG) was used to identify genes potentially involved in zinc efflux in the genome of *B. ovis* 63/290.^1^ Nucleotide sequences of the genes and the amino acid sequences of the corresponding encoded proteins were obtained from the KEGG gene database^2^ and was also used to identify orthologs and paralogs of the identified genes.

**Table 2:** Primers used in this study.

Primer name	Nucleotide sequence 5′-3’^a^	Target gene or plasmid^b^
Construction of *Brucella ovis* PA mutants		
0501MUT-F	GAACGGCTGAAGATCGAG	*0501 (BOV_0501)*
0501OVL-R	CTTCACATCGGCATGTTC	*0501 (BOV_0501)*
0501OVL-F2	gaacatgccgatgtgaagGCGAAGGAACTAATCCAT	*0501 (BOV_0501)*
0501MUT-R	TGCTTGCGTTGCTTATGC	*0501 (BOV_0501)*
0501com-F	TCTATAATGGGAGGATGC	*0501 (BOV_0501)*
0501com-R	GCTATCAGGCAGCACTCA	*0501 (BOV_0501)*
zntRMUT-F	TCCATTGCGCTCCAGAAA	*zntR (BOV_1941)*
zntROVL-R	CGGGATGCTGACCATGAT	*zntR (BOV_1941)*
zntROVL-F	atcatggtcagcatcccgCTGCACCCCACGCATTAG	*zntR (BOV_1941)*
zntRMUT-R	ACAGGCAGTGCGGTAAAA	*zntR (BOV_1941)*
zntAMUT-F3	AATCCACCGCAAACCCCA	*zntA (BOV_1942)*
zntAOVL-R	GATTTTCGCGGCACAGGA	*zntA (BOV_1942)*
zntAOVL-F3	tcctgtgccgcgaaaatcCCCTAGACGAAAGGGAAA	*zntA (BOV_1942)*
zntAMUT-R5	TGGCTGGTGTTCGGAAAA	*zntA (BOV_1942)*
zntAcom-F	AAGAGGGATATCAAAGAC	*zntA (BOV_1942)*
zntAcom-R	CTAGCGTACAGGTTGTCT	*zntA (BOV_1942)*
A1100MUT-F	CCCACCGCCAAGATTTAT	*A1100 (BOV_A1100)*
A1100OVL-R	GAGTACCATCGCCATACG	*A1100 (BOV_A1100)*
A1100OVL-F	cgtatggcgatggtactcGGGAACAGGGAGAAATAG	*A1100 (BOV_A1100)*
A1100MUT-R	GGCAAGACAACGTTTCTG	*A1100 (BOV_A1100)*
A1100com-F	TTGTCGCAATCGACAGGA	*A1100 (BOV_A1100)*
A1100com-R	TGGAAACCGGCACCTTCA	*A1100 (BOV_A1100)*
Primers for RT-qPCR or verification of recombinant plasmids and mutants		
Universal-F	GTTTTCCCAGTCACGAC	Cloning vectors
Universal-R	CAGGAAACAGCTATGAC	Cloning vectors
16S-RT Fw	TCTCACGACACGAGCTGACG	*16S (BOV_1586)*
16S-RT Rv	CGCAGAACCTTACCAGCCCT	*16S (BOV_1586)*
0501MUT-F2	AACTGTCCGCCGCATGAA	*0501 (BOV_0501)*
0501MUT-R2	GAACGTGTACACCCACTT	*0501 (BOV_0501)*
0501-R	GACGTTCAGGCTGTCATC	*0501 (BOV_0501)*
0501RT-F	TGGGAATGGGTCGATTCA	*0501 (BOV_0501)*
0501RT-R	GATTGCAGCTTCGAGCTT	*0501 (BOV_0501)*
zntRMUT-F2^c^	CGCTTTCGCCTGAAATGA	*zntA* and *zntR*
zntRMUT-R2	GTCAGTGGGCCGAACTAT	*zntR (BOV_1941)*
zntRRT-F	TTTGTTCATCCGCCATGC	*zntR (BOV_1941)*
zntAMUT-F4^c^	GAAGCTCCACTTCTGTCA	*zntA* and *zntR*
zntAMUT-R	TTAATGGTCGGAACGACA	*zntA (BOV_1942)*
zntART-F	CGACCCTCGTTCCCAAGA	*zntA (BOV_1942)*
zntASec-F1	CTTGCCGCTTCCCTTGAT	*zntA (BOV_1942)*
zntASec-F2	GCGGAAATGGTTGATCTG	*zntA (BOV_1942)*
A1100MUT-F2	TCTGCAATTGGCAGGTTC	*A1100 (BOV_A1100)*
A1100MUT-R2	CTCGATGTACGCAACCTA	*A1100 (BOV_A1100)*
A1100-R	CCGAACCAATCGCTGAAT	*A1100 (BOV_A1100)*
A1100RT-F	GCGACCGTCAACCAGAAT	*A1100 (BOV_A1100)*
znuART-F	AAGCCCATTGATACGCTG	*znuA (BOV_A1027)*
znuART-R	ATGTGCGTCATGCTCTTC	*znuA (BOV_A1027)*

a Primers were purchased from IDT, Leuven, Belgium. Lowercase sequences in 0501OVL-F2, zntROVL-F, zntAOVL-F3, and A1100OVL-F correspond to regions overlapping with 0501OVL-R, zntROVL-R, zntAOVL-R, and A1100OVL-R, respectively.

b Target gene is the *B. ovis* gene to be deleted or PCR-amplified. Primers were designed according to the published genome sequence of *B. ovis* 63/290 (ATCC 25840; accession numbers NC_009505 and NC_009504 for chromosome I and II, respectively). Primers targeting *16S* were those previously described (17). Primers Universal-F and Universal-R were used for sequencing the DNA insert of the pGEM^®^-T Easy or pCR^®^-Blunt recombinant plasmids.

c Primer zntRMUT-F2 annealed inside *zntA* and was used for verification of the Δ*zntA* and Δ*zntR* mutants and for RT-qPCR of *zntA*. Primer zntAMUT-F4 annealed inside *zntR* and was used for verification of the Δ*zntA* and Δ*zntR* mutants and for RT-qPCR of *zntR*.

DNA similarity searches were performed with the Basic Local Alignment Search Tool (BLAST)^3^ and at the Bacterial and Viral Bioinformatics Resource Center (BV-BRC).^4^ Pairwise sequence alignments of DNA or protein sequences were performed with EMBOSS Needle^5^ at the European Bioinformatics Institute. Gene Construction Kit (GCK 4.5; Textco Biosoftware, Raleigh, NC, United States) was used for the analysis of nucleotide sequences and schematic drawing of the studied loci. Protein features were retrieved from UniProt Knowledgebase (18),^6^ transmembrane topology was also analyzed with Phobius^7^ at the Stockholm Bioinformatics Center, and PSORTb v3.0.2 (Brinkman Laboratory, Simon Fraser University, British Columbia, Canada)^8^ was also used to predict protein subcellular localization (19).

For high-fidelity PCR reactions to be cloned into pCR®-Blunt, the NZYProof 2x Green Master Mix was used (NZYTech Lda., Lisboa, Portugal), while those to be cloned into pGEM®-T Easy were amplified with the Expand™ Long Template PCR System (Roche, Mannheim, Germany). The Red Taq DNA polymerase master mix (VWR, Leuven, Belgium) was used for verification of the *B. ovis* recombinant strains obtained in this study.

Relative quantification of RNA transcripts was performed by real-time reverse transcription PCR (RT-qPCR) as previously described (20). In brief, RNA was extracted, with the E.Z.N.A.^®^ Bacterial RNA Kit (Omega Bio-tek Inc., Norcross, GA, United States), from *B. ovis* strains cultured for 16 h in TSB-YE-HS, supplemented with 1 mM ZnCl_2_ or 0.05 mM CdCl_2_ when desired. RNA (1 μg) treated with RNase-free DNaseI (Thermo Fisher Scientific, Vilnius, Lithuania) to remove contaminant DNA was used to synthesize cDNA with the RevertAid H minus first strand cDNA synthesis kit (Thermo Scientific, Vilnius, Lithuania). A reaction lacking retrotranscriptase was used as a control of DNA absence. The cDNAs were submitted to real-time PCR reactions in a StepOnePlus^™^ apparatus (Applied Biosystems, Foster City, United States) with primer pairs listed in Table 2 and Express SYBR^®^ GreenER^™^ qPCR Supermix with Premixed ROX (Invitrogen, Life Technologie Corp, Carlsbad, CA, United States). Primer pairs used to evaluate the expression of each gene were as follows: 16SRT-Fw + 16SRT-Rv for *16S*, 0501RT-F + 0501RT-R for *BOV_*0501, zntRRT-F + zntAMUT-F4 for *zntR*, zntART-F + zntRMUT-F2 for *zntA*, A1100RT-F + A1100-R for *BOV_A1100,* and znuART-F + znuART-R for *znuA*. Relative expression, considering *B. ovis* PA cultured in TSB-YE-HS and *16S* as reference strain and gene (17), was evaluated with the 2^−ΔΔCt^ method using the StepOne^™^ Software v2.3. Three independent biological samples with three technical replicates were evaluated for each gene, strain, and culture condition. Results are expressed as mean ± SD of the log_2_ of the relative quantity (log_2_RQ), and statistical analysis was performed on ΔCt values (21).

### 2.3 Construction of the *Brucella ovis* recombinant strains

The procedure followed for the construction of the non-polar in-frame deletion mutants was previously described (22). In brief, the 5′ and 3` ends of each target gene, together with 300–700 bp located upstream or downstream the gene, respectively, were amplified by PCR. The primer pairs used were as follows: 0501MUT-F + 0501OVL-R and 0510OVL-F2 + 0501MUT-R for *BOV_0501,* zntRMUT-F + zntROVL-R and zntROVL-F + zntRMUT-R for *zntR,* zntAMUT-F3 + zntAOVL-R and zntAOVL-F3 + zntAMUT-R5 for *zntA*, and A1100MUT-F + A1100OVL-R and A1100OVL-F + A1100MUT-F for *BOV_A1100*. The two PCR products obtained for each gene were fused through the overlapping region existing in primers OVL-F and OVL-R, performing an additional PCR reaction with primers MUT-F + MUT-R of each gene (Table 2). The overlapping PCR reactions removed most of the DNA sequence corresponding to each target gene while maintaining the adjacent upstream and downstream regions that allow the recombination events required to produce each in-frame deletion mutant. The resulting fragments were cloned into pCR^®^-Blunt or pGEM^®^-T Easy, their nucleotide sequence determined to exclude potential undesired mutations that occurred during PCR amplification, and then subcloned into pCVD-KanD. The resulting plasmids pNV*0501*02, pNV*zntR*02, pNV*zntA*st02, and pNV*A1100*02 were electroporated into parental *B. ovis* PA, and strains with each recombinant plasmid integrated into the chromosome through a first recombination event were selected in TSA-YE-HS-kanamycin plates. These intermediate strains contain one copy of the wild-type gene and one copy of the inactivated gene. Selection of the desired mutant strains was performed by platting the intermediate strains on TSA-YE-HS-sucrose, which favors the detection of bacterial colonies suffering a second recombination event through the regions of homology located upstream or downstream of the target gene. Differentiation between colonies reverting to the wild-type genotype and the desired mutant strains was performed with PCR reactions with a pair of primers located externally to both sides of the region involved in recombination (smaller size in the mutant strain) and with primers MUT-F of each gene and a primer annealing inside each target gene (no amplification with the mutant strain).

To clone each entire gene into pBBR1MCS4, PCR amplification was performed with primers comF+comR (Table 2), and the obtained fragments were first cloned into pCR^®^-Blunt. After verification of the nucleotide sequence, each gene was excised by digestion with appropriate restriction enzymes and subcloned into pBBR1MCS4 digested with the same enzymes. The resulting plasmids pNV*0501*com, pNV*zntA*_PA_com5, pNV*zntA*_2308_com5, and pNV*A1100*com were electroporated either into parental *B. ovis* PA or into selected mutant strains (Table 1), and the recombinant strains selected and maintained in culture medium containing carbenicillin.

### 2.4 Protein techniques

Protein profiles of whole-cell bacterial lysates were obtained by sodium dodecyl sulfate-polyacrylamide gel electrophoresis (SDS-PAGE), followed by Coomassie blue staining (23). Proteins were separated in 14% acrylamide/bisacrylamide gels using a Protean II xi cell (Bio-Rad, Hercules, CA, United States) and pre-stained protein marker VI (Applichem-Panreac, Barcelona, Spain) as a protein standard.

The proteomic analysis of SDS-PAGE protein bands was performed in the proteomics facility of Centro de Investigación del Cáncer, Salamanca, Spain, following its standardized procedures. In brief, selected protein bands were excised from the gels and trypsin-digested proteins were submitted to reversed-phase LC–MS/MS using a nano-UHPLC system (NanoElute, Bruker Daltonics, Germany) coupled to a hybrid trapped ion mobility-quadrupole time-of-flight mass spectrometer Tims TOF Pro (Bruker Daltonics, Germany) via a modified nano-electrospray ion source (Captive Spray, Bruker Daltonics, Germany). Protein identification was done by searching the MS/MS spectra against the *Brucella ovis* Uniprot proteome database with the Andromeda algorithm (24) and the MAXQUANT (25). Protein relative abundance was compared using the iBAQ score (26).

### 2.5 Evaluation of virulence in macrophages and mice

Internalization and intracellular replication of the mutant strains in phagocytic cells were evaluated in J774A.1 murine macrophage as previously described (27). In brief, macrophages fixed on the surface of 96-well sterile plates were infected with each *B. ovis* strain at a multiplicity of infection of 200 CFU/macrophage. After a period of incubation of 2 h, gentamycin-containing medium (50 μg/mL) was added to the wells to kill extracellular bacteria. Intracellular bacteria were determined in three wells per strain after the lysis of macrophages by plating serial dilutions of the well content on TSA-YE-HS (t0). Intracellular bacteria were also determined in three wells per strain after 24 and 48 h of incubation (t24 and t48) in culture medium containing 20 μg/mL gentamycin. The results were expressed as means ± SD (*n* = 3) of the log_10_ CFU/well values at each time point.

Virulence in the mouse model was evaluated as previously described (28). In brief, 6-week-old BALB/c mice (Charles River Laboratories, Chatillon-sur- Chalaronne, France) were intraperitoneally infected with 10^6^ CFU of each bacterial strain. Splenic bacterium accounts were determined in five mice per group at 3 and 7 weeks post-infection (p.i.), which in *B. ovis* PA correspond to the peak of infection in the acute phase and chronic phase of infection (29). Results were expressed as means ± SD (*n* = 5) of log_10_ CFU/spleen values at each time point. The mouse experiments were designed according to the Spanish and European legislation for research with animals (RD 53/2013 and directive 2010/63/EU).

### 2.6 Statistical analysis

Statistical comparisons were performed on GraphPad Prism Software (GraphPad Software Inc., San Diego, CA, United States) with one-way ANOVA, followed by Tukey’s test or a two-tailed t-test for multiple or single comparisons, respectively. Statistically significant differences (*p* ≤ 0.05) were established with a 95% confidence interval.

## 3 Results

### 3.1 Gene and protein sequences of the ZntA-ZntR zinc efflux system of *Brucella ovis* PA and other predicted zinc exporters revealed differences between *Brucella ovis and Brucella abortus*

Using the KEGG gene and genome databases, a search for genes potentially related to zinc export was performed in the genome of *B. ovis* 63/290 (ATCC 25840) reference strain. The search confirmed that the *zntR-zntA* locus, involved in Zn^2+^ export and virulence in *B. abortus* 2308 (14), was also present in *B. ovis*, being ZntA defined as a Zn^2+^/Cd^2+^-exporting ATPase. In addition, two other genes potentially encoding proteins that might be related to zinc export were identified in the genome of *B. ovis* 63/290 reference strain: (i) the product of gene *BOV_0501*, defined as cobalt-zinc-cadmium efflux system protein, and (ii) a ZntA paralog described as a cadmium-translocating P-type ATPase encoded by *BOV_A1100*.

**Figure 1:**
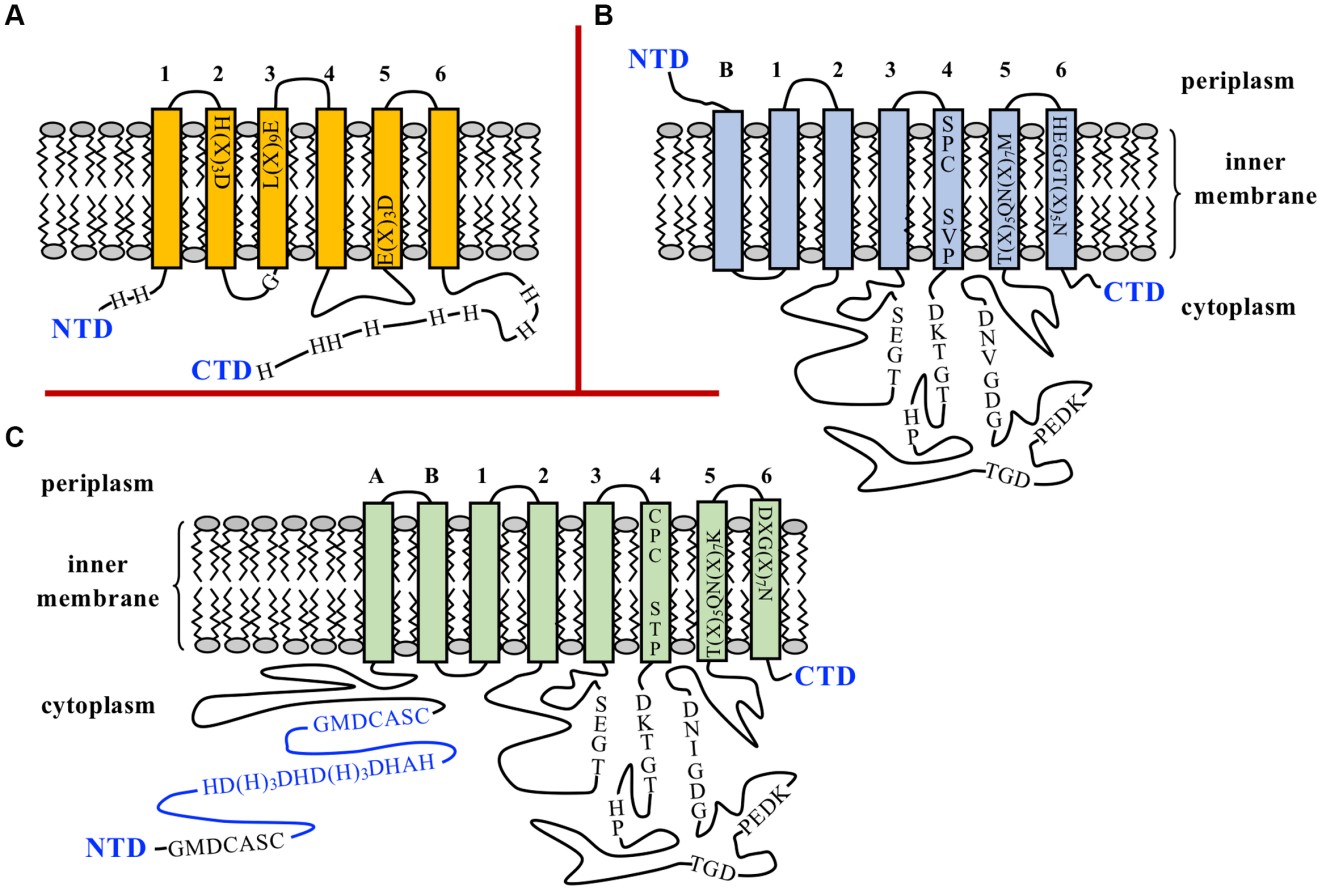
Schematic representation of BOV_0501 **(A)** and BOV_A1100 **(B)** inner membrane proteins of *Brucella ovis* PA and the ZntA Zn^2+^ exporter of *B. abortus* 2308 **(C)**. N- and C-terminal domains (NTD and CTD) are shown, and the predicted transmembrane domains (TM) are numbered. In BOV_A1100 and ZntA, the 6 TM domains that are found in all P_1B_-ATPases are numbered 1 to 6, while the N-terminal TM regions that can be present or not depending on the subgroup of P_1B_-ATPases are identified as A or B (30). In panel **(A)** the transmembrane motifs, the Gly residue adjacent to the cytoplasmic side of TM3, and the His residues of the NTD and CTD of BOV_0501, usually found in metal exporters of the cation diffusion facilitator (CDF) family (31–33), are shown. In panels **(B,C)** the characteristic motifs conserved in the actuator and nucleotide-binding domains of P_1B_-ATPases are shown in the long cytoplasmic domains connecting TM2 with TM3 and TM4 with TM5 (30, 34–36). Motifs located within TM4-TM6 and that are related to the metal specificity-selectivity of the transporter are also represented (30, 37–39). In panel **(C)** the two GXXCXXC motifs and the His-rich region, defined as metal binding motifs (40–44), are marked in the cytoplasmic NTD of *B. abortus* 2308 ZntA, and the region of the NTD that is absent in the *B. ovis* ZntA is drawn in blue (see also Figure 2).

*BOV_0501* is predicted to encode a cytoplasmic membrane protein of 297 amino acids containing six transmembrane (TM) domains, lacking signal peptide, and belonging to the cation diffusion facilitator (CDF) transporter family (Figure 1A). *BOV_A1100* is predicted to encode a P-type ATPase of 646 amino acids located at the cytoplasmic membrane, with seven TM domains and lacking signal peptide (Figure 1B). Both proteins exhibit characteristic motifs of CDF and P_1b_-type ATPase exporters, respectively, which are marked in Figure 1. DNA sequencing of *BOV_0501* and *BOV_A1100* loci in *B. ovis* PA, used as parental strain in this study, showed that both genes were identical to those of the *B. ovis* reference strain (data not shown). On the contrary, *BOV_0501* and *BOV_A1100* orthologs are annotated as pseudogenes in *B. abortus* 2308, despite the fact they share 98 and 99.7% of identity with the respective *B. ovis* genes. The reasons for these annotations are a frameshift mutation close to the 5′-end of the *BOV_0501* ortholog (*BAB1_0523*) due to a 17-bp deletion in the *B. abortus* 2308 genome and one nucleotide substitution in the *BOV_A1100* ortholog (*BAB2_1160*) resulting in a premature translation stop codon that truncates the *B. abortus* 2308 protein at amino acid 275 of 646 total amino acids (Supplementary Figure S1). The 17-bp deleted region in the *BOV_0501* ortholog of *B. abortus* 2308 is flanked in *B. ovis* by direct repeats of 6 bp that could be involved in the deletion process by a slipped mispairing mechanism (49) (Supplementary Figure S1). However, it must be noted that a potential ATG translation start codon, from which a protein of 285 amino acids could be synthesized, and is present at the *BAB1_0523* locus (Supplementary Figure S1). Therefore, the possibility of a functional BAB1_0523 protein in *B. abortus* 2308 cannot be discarded.

**Figure 2:**
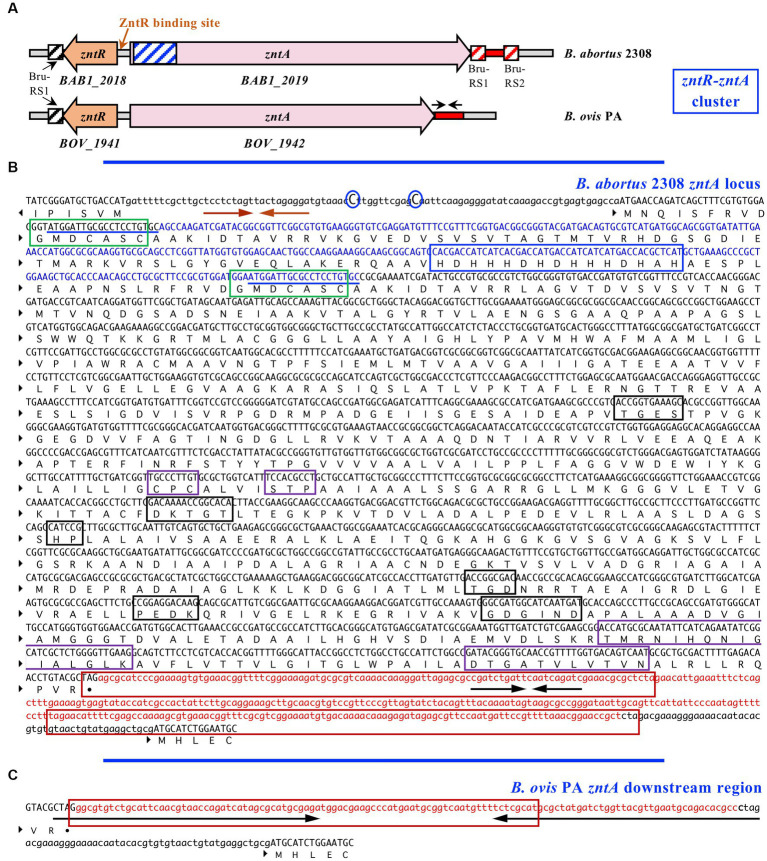
Genetic organization of the ZntR-ZntA zinc export system in *B. ovis* PA and *B. abortus* 2308 **(A)**; the nucleotide sequence of *B. abortus* 2308 *zntA* and flanking regions **(B)**; and *zntA* downstream region of *B. ovis* PA **(C)**. In panel **(A)** the predicted DNA binding site of the ZntR transcriptional regulator is marked, the 300 nt region of *B. abortus* 2308 *zntA* absent in *B. ovis* PA is represented in blue, the *zntA* downstream region differing between *B. ovis* PA and *B. abortus* 2308 is represented in red, and location of the Bru-RS1 and Bru-RS2 *Brucella* repetitive elements (45) is represented with boxes. In panel **(B)** the direct repeats probably involved in the deletion process of the 300 nt absent in *zntA* of *B. ovis* PA (blue region) are underlined. The GXXCXXC and His-rich motifs of ZntA, which could be involved in Zn binding (40–44), are framed in green and blue, respectively. Characteristic motifs located within the cytoplasmic domains of P_1B_-type ATPases (30, 34–36) are framed in black, and those identified in the transmembrane domains of P_1B-2_ ATPases are framed in purple (30, 37–39). The inverted repeats that could be involved in ZntR binding (46) and the two nt differences (C → T) between *B. abortus* 2308 and *B. ovis* PA located in the *zntR-zntA* intergenic region are marked with orange arrows and blue circles, respectively. Bru-RS1 and Bru-RS2 elements (45) downstream *zntA* in *B. abortus* 2308 are framed in red. Black arrows mark the inverted repeats detected downstream *zntA* in *B. abortus* 2308 (10 nt) and *B. ovis* PA (48 nt), which are located inside regions differing in sequence between both strains (red sequences). In panel **(C)** the 84 nt of the *zntA* downstream region identical to the 3′-end of IS*711* (47, 48) is framed.

Regarding the *zntR-zntA* locus (*BOV_1941* and *BOV_1942*, respectively), *zntR* is predicted to encode a transcriptional regulator of 140 amino acids belonging to the MerR family and with cytoplasmic localization. Minor differences between *B. ovis* PA and *B. abortus* 2308 were detected in *zntR* (99.8% identity with only one nt substitution that does not modify the encoded protein, data not shown) or in the intergenic *zntR-zntA* region (2 nt substitutions) that contains inverted repeats of 9 nt that could be involved in ZntR binding (46) (Figure 2B). The ZntA proteins of *B. abortus* 2308 and *B. ovis* PA are predicted to be P_1B_-type ATPases, lacking signal peptide, and located at the cytoplasmic membrane with 8 TM domains (Figure 1C). However, relevant differences between both strains were identified in *zntA* since they only share 86.9% identity, and an in-frame deletion of 300 bp was evidenced in the *B. ovis* PA gene (Figure 2). In the *zntA* gene of *B. abortus* 2308 (*BAB1_2019*), two direct repeats of 20 bp flank the fragment absent in *B. ovis* PA (Figure 2B), which suggests that the *B. ovis* PA deletion probably occurred through a slipped mispairing mechanism involving both repeats (49). Although it is an in-frame deletion, 100 amino acids are lost in the cytoplasmic N-terminal domain (NTD) of *B. ovis* PA ZntA, which could have an important effect on the activity of the protein (Figures 1C, 2A,C). One GXXCXXC motif, reported as Zn^2+^ binding domain in other bacteria (40–43), and a histidine-rich domain also described as a potential target for Zn binding (41–44) are found in the ZntA region of *B. abortus* 2308 (Figure 2B) that is absent in *B. ovis* PA. However, an additional GXXCXXC motif and several other motifs located in the last three TM domains that have been reported as involved in the metal translocation pathway of P_1B_-ATPases (34, 41) are conserved in both *B. ovis* PA and *B. abortus* 2308 ZntA (Figures 1C, 2B). Six additional single nt differences (accounting for four amino acid substitutions) were detected in *zntA,* but they did not result in any translational stop codon.

Moreover, the intergenic region between *zntA* and downstream *BOV_1943* (*BAB1_2020* in *B. abortus* 2308) completely differed between *B. ovis* and *B. abortus*, being constituted in *B. ovis* PA by long inverted repeats of 48 bp spaced by 31 bp (Figures 2B,C). This region is specific for *B. ovis* strains since, according to similarity searches performed at BV-BRC, the whole sequence is not found in other *Brucella* species. Although the mechanism underlying these differences is not evident, 84 bp of this *B. ovis* PA intergenic sequence (Figure 2C) shows 100% identity with the 3′-end of the insertion sequence IS*711*, which is more frequently repeated in *B. ovis* than in *B. abortus* (47, 48), while the corresponding region in *B. abortus* 2308 contains one copy of Bru-RS1 and one copy of Bru-RS2 of 103 and 106 bp, respectively, spaced by 155 bp (Figures 2A,B). Bru-RS1 and Bru-RS2 are two related repetitive palindromic elements with similar occurrence among the *Brucella* species (45). Accordingly, recombination events involving IS*711*, Bru-RS1, and/or Bru-RS2 could be responsible for these differences. It must be noted that one copy of Bru-RS1 is also present in both *B. abortus* 2308 and *B. ovis* preceding the *zntR-zntA* locus (Figure 2A).

### 3.2 ZntA is an efficient Zn^2+^ exporter in *Brucella ovis* PA and ZntR is required for maximum *zntA* expression in response to toxic zinc levels

**Figure 3:**
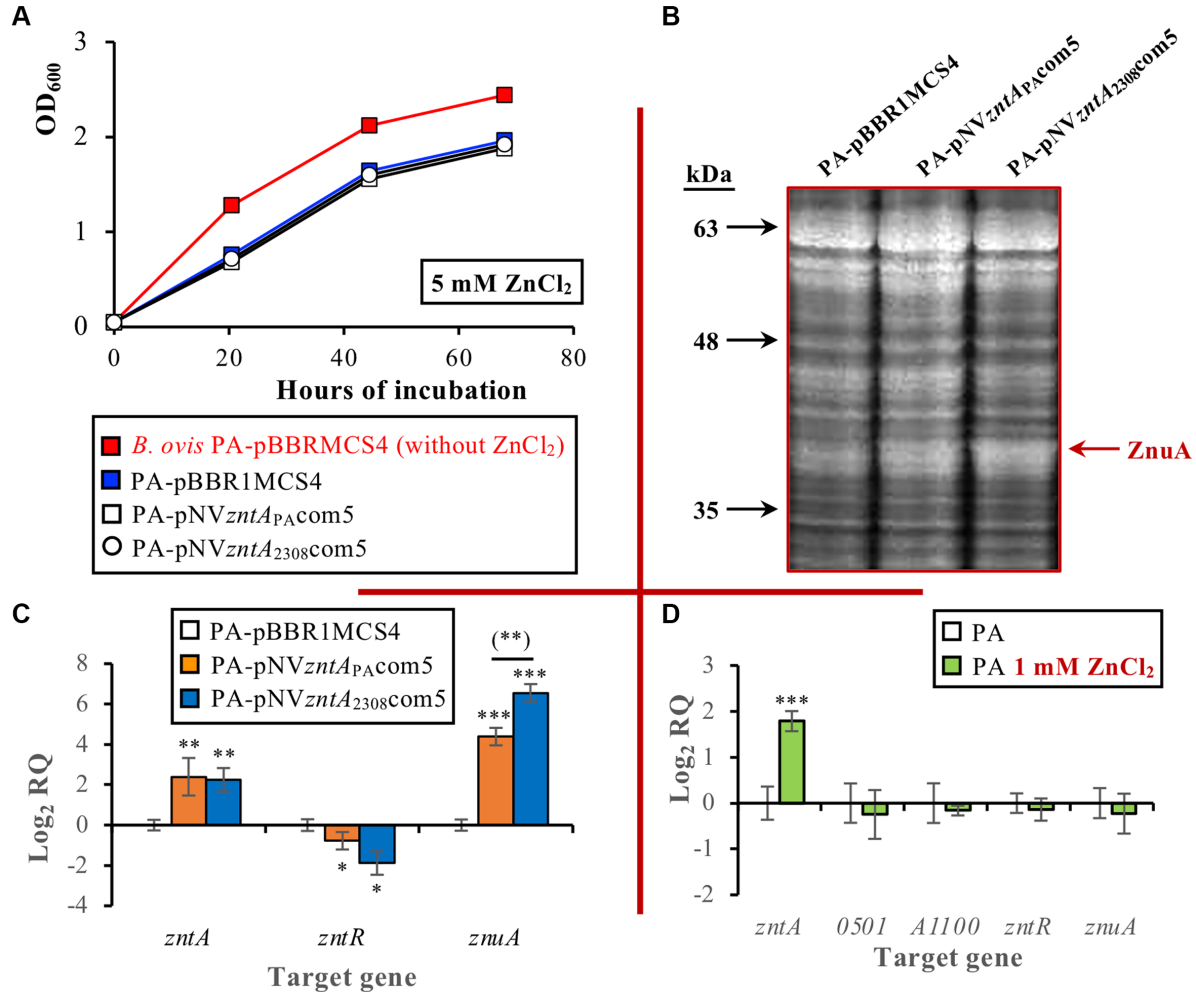
Growth curves of parental *B. ovis* PA transformed with empty pBBR1MCS4 or with pNV*zntA*_PA_com5 or pNV*zntA*_2308_com5 in the presence of 5 mM ZnCl_2_ **(A)**, proteins of the same strains separated by SDS-PAGE and stained with Coomassie blue **(B)**, and gene expression in the three strains grown in normal medium **(C)**, and of *B. ovis* PA cultured in the presence or absence of 1 mM ZnCl_2_ **(D)**. In panel **(A)** all strains exhibited the same behavior in the normal medium as that shown in red for *B. ovis* PA-pBBR1MCS4, which was also same as that of parental *B. ovis* PA (data not shown). In panel **(C)** statistically significant differences (*p* ≤ 0.05) when compared to *B. ovis* PA-pBBR1MCS4 strain are marked with asterisks, and differences between *B. ovis* PA-pNV*zntA*_PA_com5 and *B. ovis* PA-pNV*zntA*_2308_com5 are marked with asterisks in brackets. In panel **(D)** statistically significant differences (*p* ≤ 0.05) when compared to *B. ovis* PA cultured in the absence of 1 mM ZnCl_2_ are marked with asterisks. *p ≤ 0.05; **p ≤ 0.01; ***p ≤ 0.001.

Considering the reported activity of ZntA as a Zn^2+^ exporter in other bacteria, including *B. abortus* 2308 (14), and to evaluate whether the remarkable differences of the *B. ovis* PA ZntA protein described above could abolish the biological activity of the protein, *B. ovis* PA strains bearing wild-type *zntA* of *B. ovis* PA or *B. abortus* cloned in pBBR1MCS4 were constructed. Surprisingly, when the *B. ovis* PA strains were incubated in the presence of 5 mM ZnCl_2_ (a concentration slightly inhibiting growth of the parental strain), overexpression of *zntA* in *B. ovis* PA-pNV*zntA*_PA_com5 or *B. ovis* PA-pNV*zntA*_2308_com5, confirmed by RT-qPCR, did not restore the growth pattern obtained in normal medium (Figures 3A,C). Remarkably, when compared to the parental strain and *B. ovis* PA-pBBR1MCS4, both strains showed in normal medium an overrepresented protein band in SDS-PAGE that, according to LC–MS/MS analysis, does not correspond to the ZntA exporter but to ZnuA (Figure 3B), a periplasmic zinc-binding protein involved in zinc import in *B. abortus* 2308 (14, 50). These results are in accordance with *znuA* overexpression detected by RT-qPCR in both strains when compared to *B. ovis* PA-pBBR1MCS4 (Figure 3C). According to SDS-PAGE results, ZnuA appeared to be more abundant in *B. ovis* PA-pNV*zntA*_2308_com5 than in *B. ovis* PA-pNV*zntA*_PA_com5 (Figure 3B), which was also in agreement with RT-qPCR results that showed that although both strains exhibited similar levels of *zntA* overtranscription (log_2_ RQ mean values of 2.4 and 2.2, respectively), *znuA* transcripts were more abundant (*p* ≤ 0.01) in *B. ovis* PA-pNV*zntA*_2308_com5 (log_2_ RQ mean value of 6.6) than in *B. ovis* PA-pNV*zntA*_PA_com5 (log_2_ RQ mean value of 4.4; Figure 3C). On the contrary, both strains exhibited slightly reduced levels of *zntR* transcripts (*p* ≤ 0.05) when compared to *B. ovis* PA-pBBR1MCS4 (Figure 3C).

An RT-qPCR assay was performed with parental *B. ovis* PA cultured with or without 1 mM ZnCl_2_ to determine the effect of exposure to toxic levels of zinc on the expression of genes related to zinc export and *znuA*. Exposure to 1 mM ZnCl_2_ increased about 3.5 times the level of transcripts of *zntA* (log_2_ RQ mean value of 1.79; *p* ≤ 0.001) when compared to *B. ovis* PA cultured in a normal medium, while no statistically significant differences were found in expression of *znuA, zntR, BOV_0501,* or *BOV_A1100* (Figure 3D).

To verify the biological activity of the *B. ovis* PA ZntA protein, the construction of the *zntA* deletion mutant was attempted in this strain. The process was challenging, probably due to the long-inverted repeats located downstream of the gene that presumably hamper the required recombination event. The *B. ovis* PA Δ*zntA* mutant could only be obtained when the inverted repeats were included as part of the *zntA* deleted region in the pNV*zntA*st02 plasmid used to obtain the mutant. The mutant strain was subsequently complemented *in trans* with either pNV*zntA*_PA_com5 or pNV*zntA*_2308_com5. The *B. ovis* PA Δ*zntR* mutant was also constructed and subsequently transformed with pBBR1MCS4, pNV*zntA*_PA_com5, and pNV*zntA*_2308_com5.

**Figure 4:**
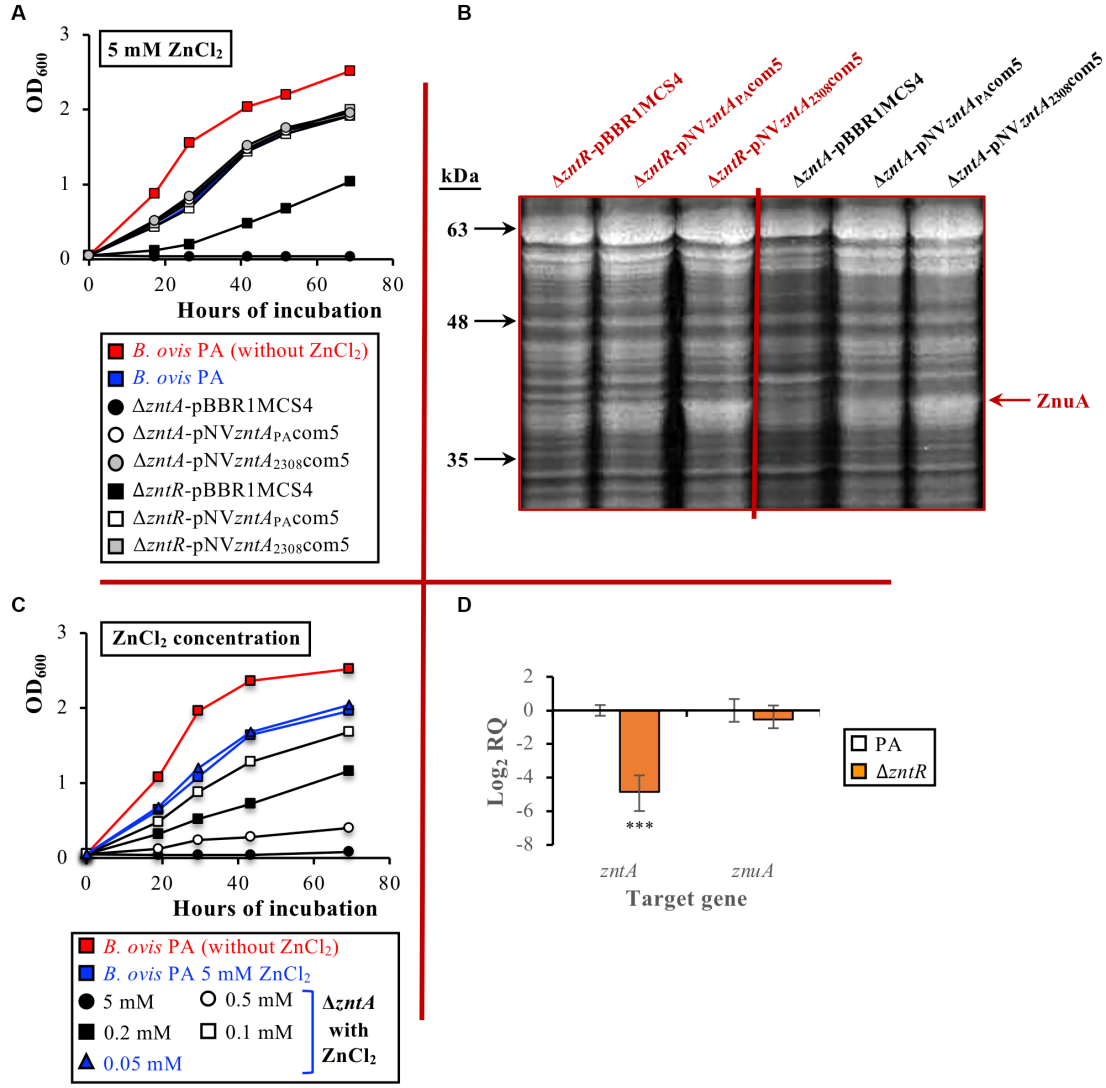
Growth curves of parental *B. ovis* PA and the Δ*zntR* and Δ*zntA* isogenic mutants transformed with empty pBBR1MCS4 or with pNV*zntA*_PA_com5 or pNV*zntA*_2308_com5 in the presence of 5 mM ZnCl_2_ **(A)**, proteins of the same strains separated by SDS-PAGE and stained with Coomassie blue **(B)**, growth curves of the Δ*zntA* mutant in the presence of several ZnCl_2_ concentrations **(C)**, and gene expression in the Δ*zntR* mutant grown in normal medium **(D)**. In panel **(A)** all strains exhibited the same behavior in the normal medium as that shown in red for *B. ovis* PA (data not shown). In panel **(C)** blue curves correspond to ZnCl_2_ concentrations, giving an equivalent growth pattern in parental *B. ovis* PA and its isogenic Δ*zntA* mutant. In panel **(D)** statistically significant differences (*p* ≤ 0.05) when compared to *B. ovis* PA are marked with asterisks. ***p ≤ 0.001.

The absence of ZntR or ZntA did not affect the growth of *B. ovis* PA in a normal medium (data not shown; the Δ*zntR* and the Δ*zntA* mutants behaved as parental *B. ovis* PA in Figure 4A). However, in the presence of 5 mM ZnCl_2_, the Δ*zntR* mutant showed a defective growth while the Δ*zntA* mutant was unable to grow (Figure 4A). Both mutants recovered the parental phenotype after transformation with pNV*zntA*_PA_com5 or pNV*zntA*_2308_com5 (Figure 4A), which suggests that growth defects of the Δ*zntR* mutant under toxic ZnCl_2_ concentrations are due to underexpression of ZntA. In fact, transcripts for *zntA* in the Δ*zntR* mutant were in the order of 30-fold lower (mean log2 RQ value of −4,86) than those detected in the parental strain (*p* ≤ 0.001), while no statistically significant differences were observed between both strains regarding *znuA* expression (Figure 4D). The low levels of *zntA* transcripts in the Δ*zntR* mutant are in accordance with both its increased sensitivity to ZnCl_2_ and the recovery of the parental phenotype in strains Δ*zntR*-pNV*zntA*_PA_com5 and Δ*zntR*-pNV*zntA*_2308_com5 (Figures 4A,D). Therefore, although the low transcription level of *zntA* in the absence of ZntR allows relevant levels of Zn^2+^ detoxification in the Δ*zntR* mutant (Figure 4A), the presence of ZntR is required in *B. ovis* PA to obtain maximum ZntA levels in response to exposure to toxic concentrations of ZnCl_2_ (Figures 4A,D). When compared to the parental strain, transcription of *BOV_0501* and *BOV_A1100* was not increased in the Δ*zntR* and Δ*zntA* mutants or in the Δ*zntA* mutant cultured in the presence of ZnCl_2_ (data not shown).

As described above for *B. ovis* PA-pNV*zntA*_PA_com5 or *B. ovis* PA-pNV*zntA*_2308_com5 (Figure 4B), complementation *in trans* of the Δ*zntA* mutant with pNV*zntA*_PA_com5 or pNV*zntA*_2308_com5 led to an increase of the ZnuA periplasmic protein related to zinc import that was perceptible in SDS-PAGE (Figure 4B). Similar results were obtained with the Δ*zntR* mutant transformed with the same plasmids (Figure 4B).

Since the Δ*zntA* mutant was unable to grow in the presence of 5 mM ZnCl_2_, several ZnCl_2_ concentrations were assayed to determine its degree of sensitivity to this compound in comparison to that of the parental strain. The Δ*zntA* mutant only recovered a growth pattern equivalent to that obtained with the parental strain incubated with 5 mM ZnCl_2_ when a ZnCl_2_ concentration 100 times lower (0.05 mM) was used (Figure 4C). With the Δ*zntR* mutant, which was more resistant to ZnCl_2_ toxicity than the Δ*zntA* mutant (Figure 4A), the parental phenotype was recovered with a ZnCl_2_ concentration 5 times lower (1 mM; Supplementary Figure S2).

### 3.3 ZntA is an efficient Cd^2+^ exporter in *Brucella ovis* PA also contributing to Co^2+^, Cu^2+^, and Ni^2+^ detoxification

**Figure 5:**
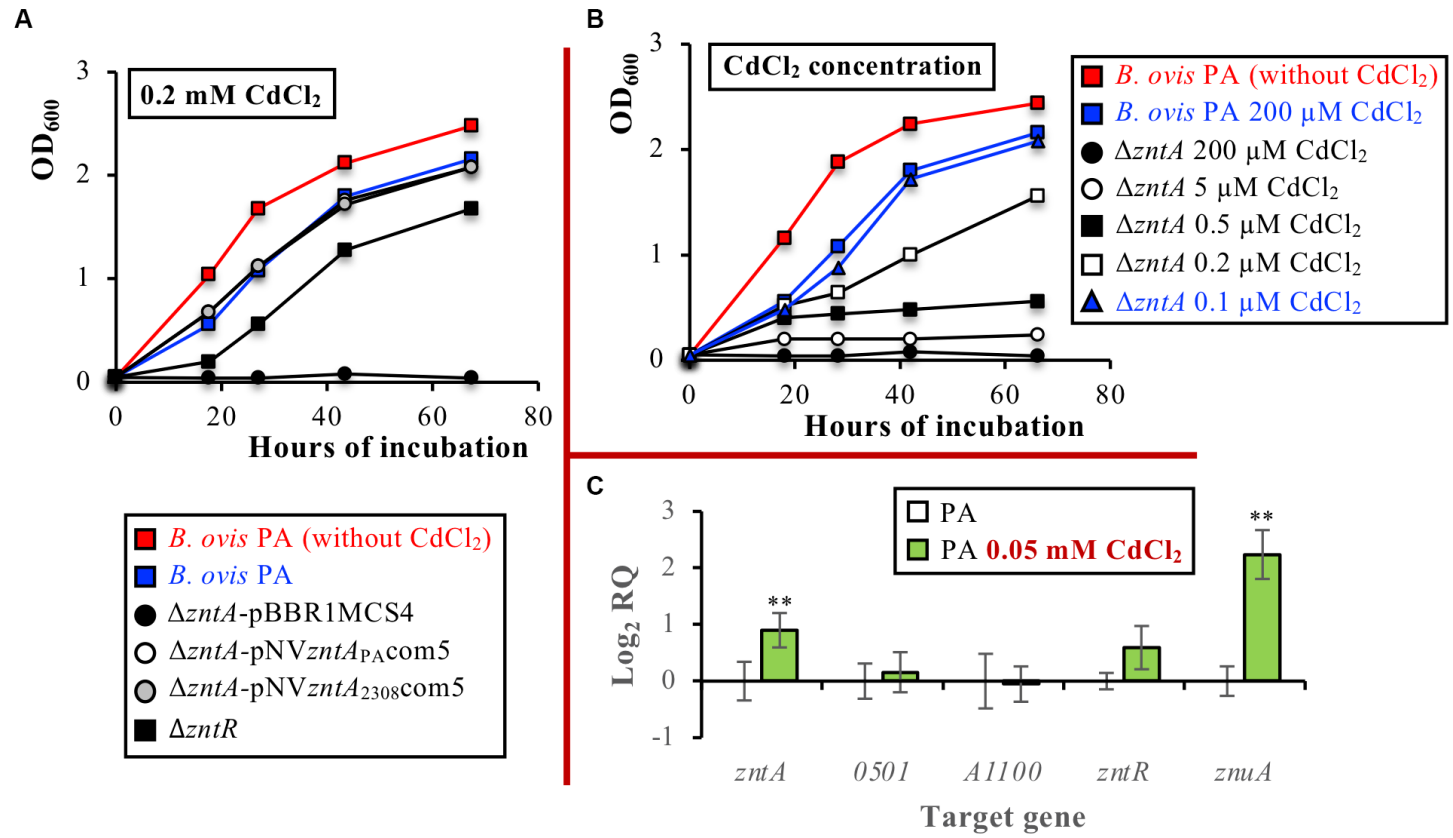
Growth curves of parental *B. ovis* PA, the Δ*zntR* mutant, and the Δ*zntA* mutant transformed with empty pBBR1MCS4 or with pNV*zntA*_PA_com5 or pNV*zntA*_2308_com5 in the presence of 0.2 mM CdCl_2_ **(A)**, growth curves of the Δ*zntA* mutant in the presence of several CdCl_2_ concentrations **(B)**, and gene expression in parental *B. ovis* PA cultured in the presence or absence of 0.05 mM CdCl_2_ **(C)**. In panel **(A)** all strains exhibited the same behavior in the normal medium as that shown in red for *B. ovis* PA (data not shown). In panel **(B)** blue curves correspond to CdCl_2_ concentrations, giving an equivalent growth pattern in parental *B. ovis* PA and its isogenic Δ*zntA* mutant. In panel **(C)** statistically significant differences (*p* ≤ 0.05) when compared to *B. ovis* PA cultured in the absence of 0.05 mM CdCl_2_ are marked with asterisks. **p ≤ 0.01.

ZntA orthologs in several bacteria have been described as relevant exporters for other toxic divalent cations (mainly Cd^2+^ but also Pb^2+^ and Co^2+^) (40, 46). Accordingly, and since this aspect has not been evaluated in any other pathogenic brucellae, the role of ZntA in resistance to Cd^2+^, Co^2+^, Pb^2+^, Hg^2+^, Mn^2+^, Fe^2+^, Cu^2+^, and Ni^2+^ was evaluated in *B. ovis* PA. Incubation with 0.2 mM CdCl_2_, a concentration slightly affecting the growth of the parental strain, impaired the growth of the Δ*zntR* mutant and prevented the multiplication of the Δ*zntA* mutant (Figure 5A). Overexpression of wild-type ZntA from *B. ovis* PA or *B. abortus* 2308 in both mutants restored the parental phenotype (Figure 5A), which reveals the role of the ZntA protein of both *B. ovis* PA and *B. abortus* 2308 as a powerful Cd^2+^ exporter. The high efficiency of ZntA in CdCl_2_ detoxification was demonstrated when growth under several CdCl_2_ concentrations was evaluated since parental *B. ovis* PA tolerated a CdCl_2_ concentration 2000 times higher than that supported by the isogenic Δ*zntA* mutant (Figure 5B). The presence of ZntR also contributed to Cd^2+^ detoxification mediated by ZntA, but tolerance of the Δ*zntR* mutant to CdCl_2_ was only twice lower than that of the parental strain (Supplementary Figure S2). Despite this demonstrated role of ZntA as a cadmium exporter, only a discrete increase of *zntA* transcription, accompanied by increased levels of *znuA* transcripts, was observed in *B. ovis* PA incubated with CdCl_2_ (*p* ≤ 0.01). The expression of *BOV_A1100* and *BOV_0501* in the presence of CdCl_2_ was similar to that observed with the parental strain (Figure 5C).

**Figure 6:**
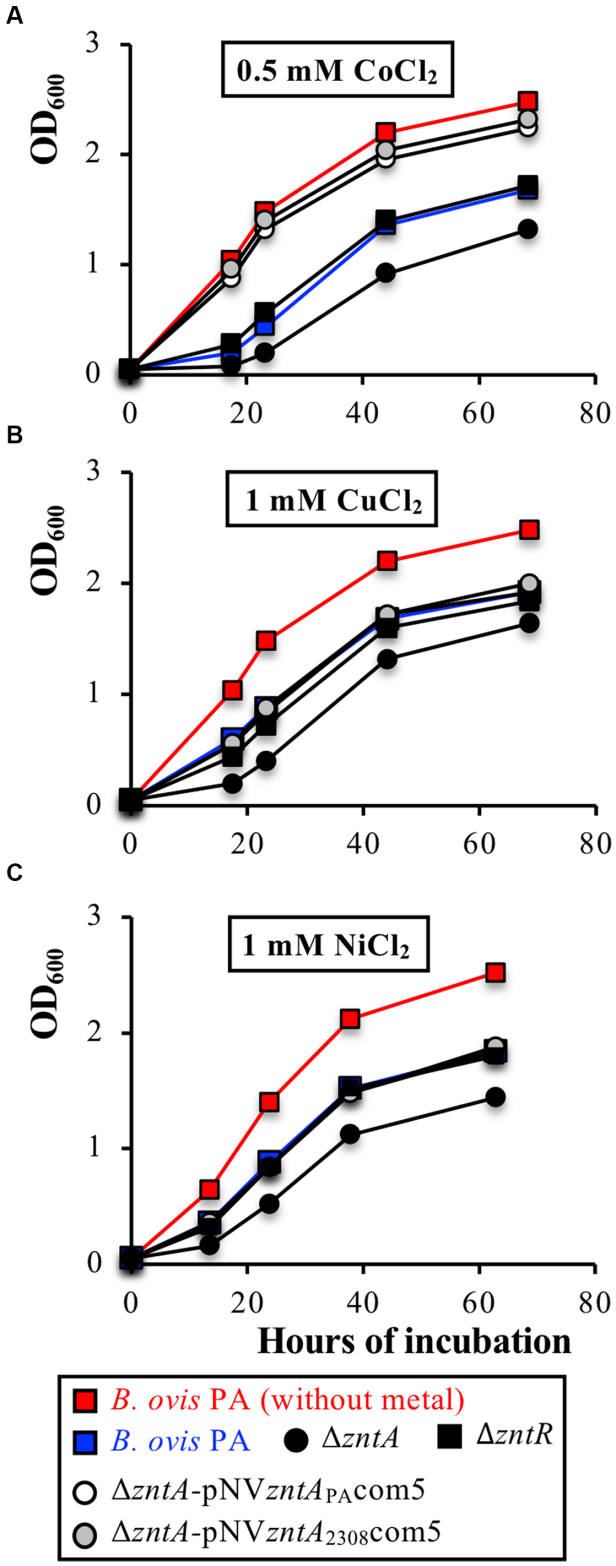
Role of the ZntR-ZntA export system in the survival of *B. ovis* PA exposed to high concentrations of CoCl_2_ **(A)**, CuCl_2_ **(B)**, or NiCl_2_ **(C)**. The growth curves of Δ*zntA-*pNV*zntA*_PA_com5 and Δ*zntA-*pNV*zntA_2308_*com5 in each panel are similar. All strains gave the same growth profile in normal medium and are not represented in the figure (see growth of *B. ovis* PA in red lanes as representative curve). Similarly, *B. ovis* PA, *B. ovis* Δ*zntR,* and *B. ovis* Δ*zntA* transformed with non-recombinant pBBR1MCS4 behaved as the respective parental strains and are not shown in the figure. In panel **(A)** parental *B. ovis* PA transformed with pNV*zntA*_PA_com5 or pNV*zntA*_230*8*_com5 also exhibited a growth pattern in the presence of 0.5 mM CoCl_2_ close to that of the parental strain in normal medium (data not shown).

The *B. ovis* PA Δ*zntA* mutant, but not the complemented strains or the Δ*zntR* mutant, also showed increased sensitivity to 0.5 mM CoCl_2_, 1 mM CuCl_2_, and 1 mM NiCl_2_ (Figure 6), while the absence of ZntA did not modify susceptibility to 0.8 mM PbCl_2_, 2 mM MnCl_2_, 2 mM FeCl_2_, or 4 μM HgCl_2_ (data not shown). Remarkably, growth with CoCl_2_ 0.5 mM of the Δ*zntA*-pNV*zntA*_PA_com5 and Δ*zntA*-pNV*zntA*_2308_com5 complemented strains was even better than that obtained with *B. ovis* PA, while with ZnCl_2_, CdCl_2_, CuCl_2_, and NiCl_2_, the three strains showed the same growth pattern (Figures 4–6).

### 3.4 BOV_0501 and BOV_A1100 have Zn^2+^ and Co^2+^ exporter activity, respectively, that is not required for Zn^2+^ or Co^2+^ detoxification in *Brucella ovis* PA when ZntA is present

Deletion mutants for *BOV_0501* and *BOV_A1100* were also constructed, and their growth was evaluated in the presence of 5 mM ZnCl_2_, 0.2 mM CdCl_2_, 0.5 mM CoCl_2_, 1 mM CuCl_2_, 1 mM NiCl_2_, 0.8 mM PbCl_2_, 2 mM MnCl_2_, 2 mM FeCl_2_, or 4 μM HgCl_2_. Both mutants always behaved as the parental strain and, therefore, no relevance for the detoxification of any of these compounds could be demonstrated for BOV_0501 and BOV_A1100 proteins under the tested conditions (data not shown).

**Figure 7:**
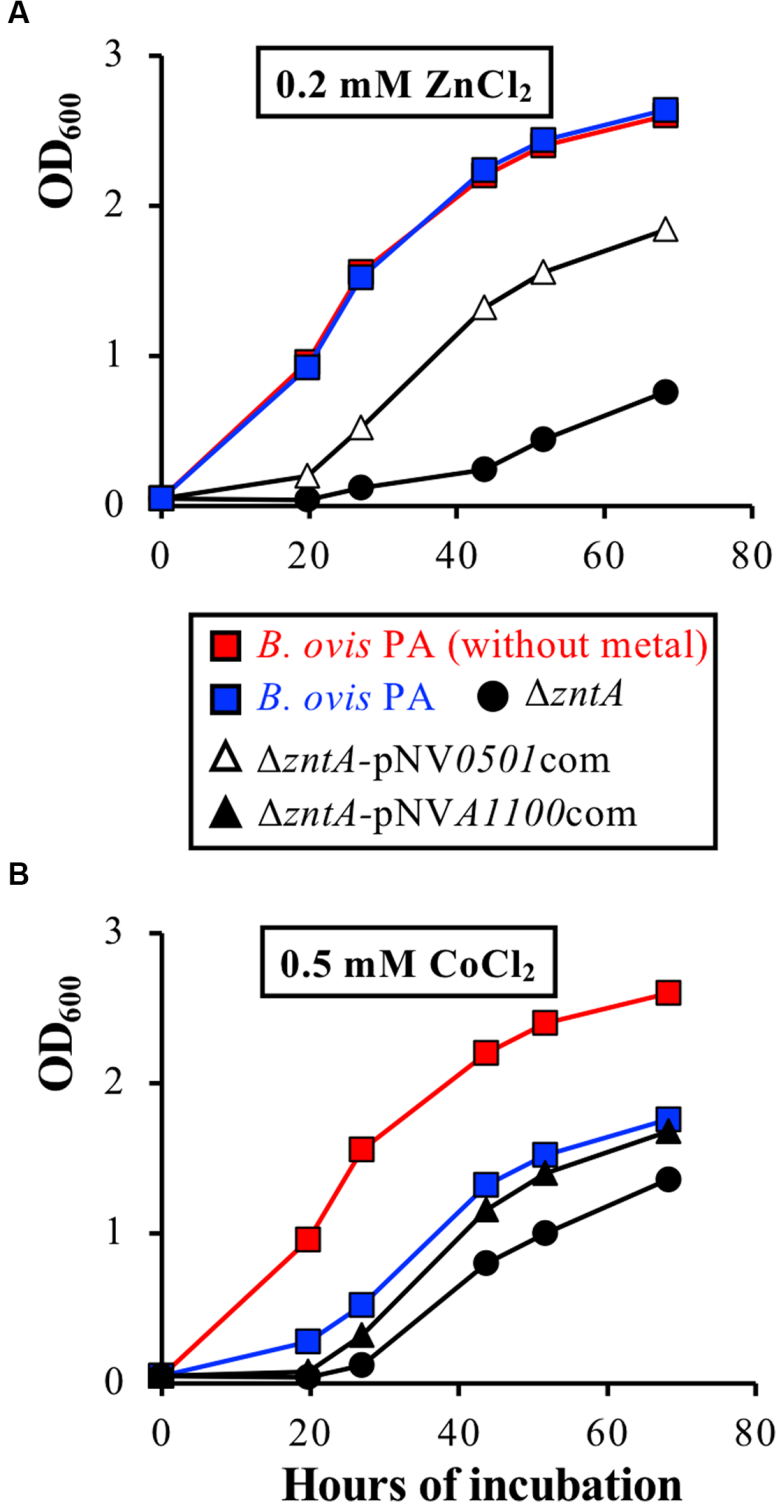
The ability of overexpression of *BOV_0501* to restore the growth defects of the *B. ovis* PA Δ*zntA* mutant in the presence of ZnCl_2_ 0.2 mM **(A)** or of overexpression of *BOV_A1100* to restore the growth defects of the *B. ovis* PA Δ*zntA* mutant in the presence of 0.5 mM CoCl_2_ **(B)**. All strains gave the same growth profile in normal medium, including strains transformed with empty pBBR1MCS4, and are not represented in the figure (see growth of *B. ovis* PA in red lanes as representative curve).

Since both genes are predicted to encode Zn^2+^ and/or Cd^2+^ exporters and considering the high efficiency of ZntA as an exporter of both cations in *B. ovis* PA, recombinant plasmids bearing wild-type *BOV_0501* or *BOV_A1100* (pNV*0501*com and pNV*A1100*com) were introduced into the Δ*zntA* mutant to evaluate whether overexpression of these genes in strains *B. ovis* Δ*zntA-*pNV*0501*com and *B. ovis* Δ*zntA-*pNV*A1100*com (Table 1) could increase the resistance of the Δ*zntA* mutant to Zn^2+^, Cd^2+^, and/or the other divalent cations analyzed in this study. Strain *B. ovis* Δ*zntA-*pNV*0501*com showed a better growth pattern in the presence of ZnCl_2_ (Figure 7A), but not in the presence of the other metals (data not shown), than that observed with the Δ*zntA* mutant. Overexpression of *BOV_A1100* in *B. ovis* Δ*zntA-*pNV*A1100*com led to increased resistance of the Δ*zntA* mutant to Co^2+^ (Figure 7B) but not to Zn^2+^ or the other divalent cations tested (data not shown).

Accordingly, although BOV_0501 and BOV_A1100 show Zn^2+^ and Co^2+^ efflux activity, respectively, their role as exporters in *B. ovis* PA does not seem relevant, at least when ZntA is present. It must be noted that transcription of both genes remained low in the parental strain incubated in normal medium or in the presence of 1 mM ZnCl_2_ or 0.05 mM CdCl_2_, as also occurred in the Δ*zntA* and Δ*zntR* mutants (data not shown).

### 3.5 ZntR, ZntA, BOV_0501, and BOV_A1100 are not required for the virulence of *Brucella ovis* PA in macrophages or mice

**Figure 8:**
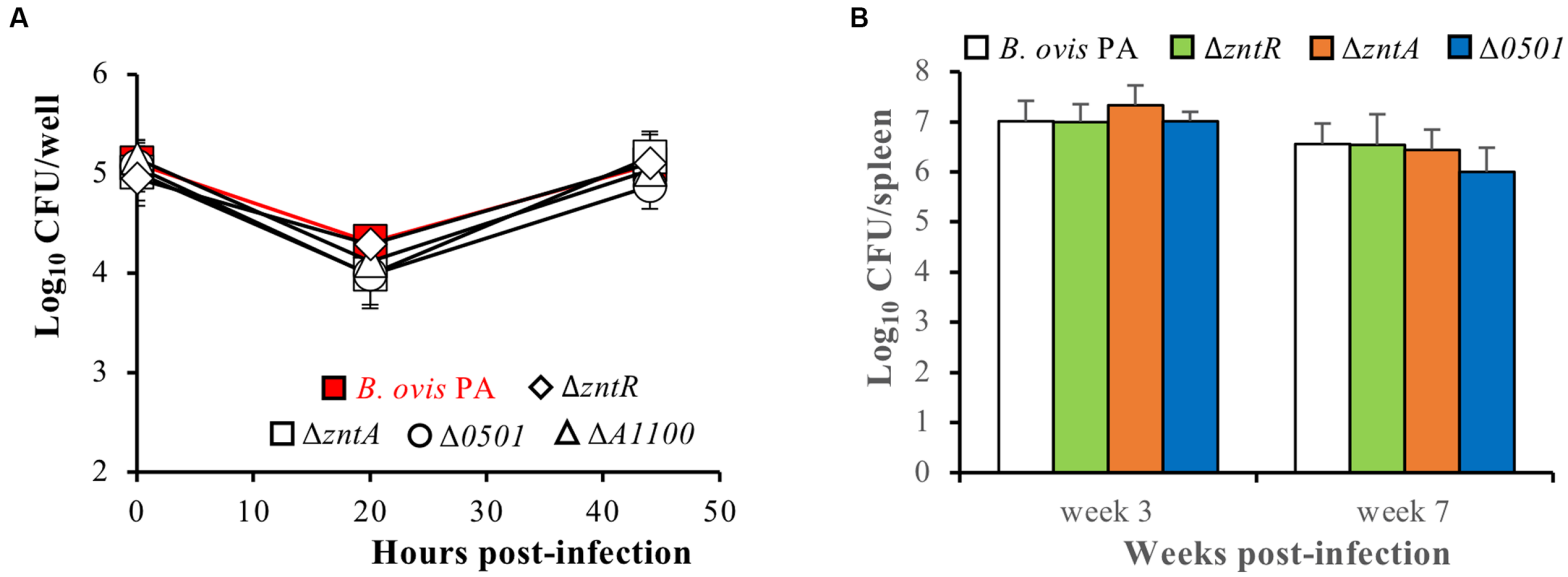
Virulence of the *B. ovis* PA deletion mutants in J774A.1 murine macrophages **(A)** and mice intraperitoneally infected with 10^6^ CFU/mice **(B)**. Intracellular bacteria in murine macrophages were enumerated at three time points after infection, and results for each strain and time point are expressed as means ± SD (*n* = 3) of the log_10_ CFU/well. Strain colonization in mice was evaluated by CFU accounts in spleen at weeks 3 and 7 post-infection, and results are expressed as means ± SD (*n* = 5) of the log_10_ CFUs/spleen. No statistically significant differences were detected in any strain compared to the parental strain.

The internalization and intracellular behavior of the deletion mutants in phagocytic cells was evaluated in J774A.1 murine macrophages. No differences were observed between the deletion mutants and the parental strain regarding internalization or intracellular replication. All strains showed intracellular numbers of about 5 log_10_ units at t0, a reduction of CFU/well in the order of 1 log_10_ unit 20 h after internalization, and intracellular replication thereafter to reach intracellular count levels at t44 that were close to those observed at t0 (Figure 8A).

Taking into account the previous results described for the ZntR-ZntA system in *B. abortus* 2308 (14) and those obtained here for all *B. ovis* PA mutants regarding gene expression, metal toxicity, and virulence in macrophages, only the Δ*zntR* and Δ*zntA* mutants were considered to merit evaluation in mice. However, since a discrete role in zinc export was detected for BOV_0501 that could be related to the differences observed in the ZntR-ZntA system between *B. ovis* and *B. abortus,* the Δ*BOV_0501* mutant was also evaluated in mice. Spleen colonization at the acute and chronic phase of infection (weeks 3 and 7 post-infection, respectively) did not show reduced virulence of the mutant strains when compared to the parental strain (Figure 8B), which contrasts with the attenuated phenotype described for the Δ*zntR* mutant of *B. abortus* 2308 (14).

## 4 Discussion

Despite zinc’s essentiality for optimal functioning of the bacterial cell, its intracellular concentration must be finely tuned to avoid toxicity that is mainly derived from the high affinity of zinc by metal binding sites, which leads to mismetallation of metalloproteins that do not require zinc but other metals (9, 11, 13). Zinc intoxication, also detected inside macrophages, is considered part of the innate immune response of the host against microbes (9, 11, 13). Therefore, bacterial pathogens have evolved zinc efflux systems of three main types to fight against host defenses: (i) P-type ATPases, (ii) cation diffusion facilitator family exporters, and (iii) resistance-nodulation-cell division (RND) transporters (11–13).

Out of these three mechanisms of zinc export, only the first one has been evidenced in the genus *Brucella*, being ZntA, a P-type ATPase exclusively studied in *B. abortus* 2308 and whose expression is transcriptionally regulated by ZntR in a Zn^2+^ dependent manner (14). In the present study, the ZntR-ZntA system has also been shown to play a prominent role in circumventing zinc toxicity in *B. ovis* PA (Figure 4), but when compared to *B. abortus* 2308, relevant differences regarding ZntA amino acid sequence and activity, regulation of its expression by ZntR, and role in virulence have been demonstrated. According to the absence of 100 amino acids in the *B. ovis* ZntA protein (Figures 1C, 2), which is one of these main differences, a loss of functionality of the protein would be conceivable. However, results presented in this study demonstrate the increased sensitivity of the *B. ovis* Δ*zntA* mutant to toxic ZnCl_2_ concentrations (Figure 4) and equivalent resistance to this compound mediated by overexpression of *B. ovis* or *B. abortus* 2308 *zntA* (Figures 3, 4), which evidences that the ZntA proteins of both *Brucella* strains are functional zinc exporters with an apparent equivalent efficiency.

In fact, both *Brucella* ZntA proteins contain the motifs that are strictly conserved across all P_1B_-type ATPases (transition and heavy metal transporters) and that are part of the cytoplasmic ATP-binding and actuator domains (Figures 1C, 2B) (30, 34–36). Additionally, their TM4, TM5, and TM6 domains contain the characteristic motifs of the subgroup P_1B-2_ of P_1B_-type ATPases, which are Pb^2+^/Zn^2+^/Cd^2+^ efflux pumps (Figure 1C) (30, 37–39). The two Cys of the CPC motif and the Asp residue located in TM4 and TM6, respectively (Figure 1C), are known to be involved in the metal specificity of ZntA-related P_1B_-type ATPases and required for coordination of Zn^2+^, Cd^2+^, and Pb^2+^ (37, 38). However, a role for ZntA in Pb^2+^ detoxification could not be demonstrated in *B. ovis*, which might be related to the amino acid sequence of the cytoplasmic NTD. In *E. coli*, the NTD of ZntA contains the GXXCXXC and CCCDGAC motifs and can bind Zn^2+^, Cd^2+^, and Pb^2+^, but the ability to bind Pb^2+^ is lost when the CCCDGAC motif is removed (51). Therefore, the presence of a canonical GXXCXXC metal binding motif (GMDCASC) and the absence of the CCCDGAC motif in the *B. ovis* ortholog (Figures 1C, 2B) could explain, at least in part, the similar behavior of parental *B. ovis* PA and its isogenic Δ*zntA* mutant in the presence of toxic PbCl_2_ levels, while the growth of the Δ*zntA* mutant was highly impaired in the presence of toxic ZnCl_2_ and CdCl_2_ levels (Figures 4, 5). Considering that the NTD of *B. abortus* 2308 ZntA contains an additional GMDCASC motif and a His-rich region, also found in some Zn^2+^/Cd^2+^ P_1B_-type ATPases (42–44), a higher Zn^2+^ export activity could be expected for this ZntA protein. However, this property could only be inferred through indirect evidence provided by the increased levels of *znuA* transcripts, and of its encoded protein, detected in *B. ovis* PA-pNV*zntA*_2308_com5 when compared to those detected in *B. ovis* PA-pNV*zntA*_PA_com5 (Figures 3B,C). Since ZnuA is a high-affinity zinc-binding periplasmic protein involved in zinc import (50), these results would be in accordance with a relevant reduction of intracellular zinc levels caused by overexpression of the ZntA zinc exporter, which would require to be counterbalanced by overexpression of zinc importer genes to maintain zinc homeostasis and cell viability.

The remarkable role of *B. ovis* ZntA in conferring resistance to cadmium (Figure 5) has not been reported before in the genus *Brucella*. However, it must be noted that this property was not evaluated with the Δ*zntA* mutant of *B. abortus* 2308 (14) and that our results confirm the powerful activity as cadmium exporters of both *Brucella* ZntA proteins (Figure 5A). In fact, the *B. ovis* PA Δ*zntA* mutant tolerates concentrations of ZnCl_2_ and CdCl_2_ in the order of 100-fold and 2000-fold lower than the parental strain, respectively (Figures 4C, 5B). Accordingly, although the presence of additional exporters contributing to Zn^2+^ efflux cannot be discarded, a higher efficiency of ZntA as a cadmium exporter could be inferred, considering that levels of *zntA* transcripts in *B. ovis* PA cultured in the presence of CdCl_2_ are not higher than those observed in the presence of ZnCl_2_ (Figures 3D, 5C). The increment of *zntA* expression in the presence of CdCl_2_ was accompanied by higher expression of *znuA* (Figure 5C), which could be related to an enhanced Zn^2+^ efflux mediated by ZntA increase. Alternatively, cadmium could compete with zinc for import, as suggested for similar results obtained with *Klebsiella pneumoniae* (52).

The results obtained in this study confirm that ZntA from both *B. ovis* PA and *B. abortus* 2308 also contributes to Co^2+^, Ni^2+^, and Cu^2+^ detoxification (Figure 6), which is in accordance with the reported ability of the three cations to act as substrates for *E. coli* ZntA, although with a much lower ATPase activity than that observed with Pb^2+^, Zn^2+^, and Cd^2+^ (40). Remarkably and in contrast to the results obtained with the other metals, overexpression of *zntA* in parental *B. ovis* PA or the Δ*zntA* mutant increased their resistance to CoCl_2_, allowing the recovery of growth patterns equivalent to those observed in normal medium (Figure 6A). Similar results have been reported in *Helicobacter pylori,* where *in trans* complementation of the Δ*cadA* mutant restored Zn^2+^ and Cd^2+^ susceptibility of the parental strain but increased the tolerance to Co^2+^ exposure (53).

Another significant difference observed in the ZntR-ZntA system between *B. abortus* 2308 and *B. ovis* refers to the ZntR-mediated regulation of *zntA* expression and the susceptibility of the Δ*zntR* mutant to ZnCl_2_. Thus, the deletion of *zntR* in *B. abortus* 2308 did not modify susceptibility to ZnCl_2_ but led to increased levels of *zntA* and *znuA* transcripts (14). ZntR was shown to bind to the *zntR-zntA* intergenic region in *B. abortus* 2308 and seemed to limit *zntA* expression under normal zinc availability conditions while it activated *zntA* expression in the presence of zinc excess, acting as a canonical MerR-type regulator (14). On the contrary, when compared to the parental strain, the *B. ovis* PA Δ*zntR* mutant was about 5-fold more susceptible to ZnCl_2_ than the parental strain (Supplementary Figure S2) and showed a marked reduction of *zntA* transcripts and unaltered *znuA* expression (Figure 4D). These results are consistent with the observation that growth defects of the Δ*zntR* mutant were abolished when *zntA* was overexpressed by transformation with pNV*zntA*_PA_com5 or pNV*zntA*_2308_com5 (Figure 4A). Accordingly, ZntR does not seem to act in *B. ovis* PA as a repressor of *zntA* expression in a normal medium but rather to be an activator required for maximal *zntA* expression in response to increased Zn^2+^ levels (Figure 4A). These differences between *B. abortus* 2308 and *B. ovis* PA are difficult to explain since both ZntR proteins are identical, but might be dependent on the two nucleotide differences observed between both strains in the *zntR-zntA* intergenic region (Figure 2B), which could favor *zntA* transcription in *B. ovis* PA in normal growth conditions.

Additionally, both *zntR* and *zntA* are dispensable for virulence of *B. ovis* PA in the mouse model (Figure 8), while the Δ*zntR* mutant of *B. abortus* 2308 is attenuated (14). The increased level of *zntA* transcripts, which would reduce zinc availability in the host, was suggested to be the cause of this attenuation. This fact, together with the probable higher efficiency as a zinc exporter of *B. abortus* 2308 ZntA (Figures 3, 4) and the underexpression of *zntA* in the *B. ovis* PA Δ*zntR* mutant (Figure 4D), could explain, at least in part, the different virulent phenotype of the two Δ*zntR* mutants. As also observed with *B. abortus* 2308 (14), the full virulence of the *B. ovis* PA Δ*zntA* mutant, despite its high susceptibility to ZnCl_2_, suggests that host-induced zinc intoxication is not a relevant mechanism for the control of infection, at least in mice. However, the presence of additional Zn^2+^ exporters that could help to control limited intracellular toxic levels cannot be excluded.

Deletion of *BOV_0501* and *BOV_A1100*, coding for the two other hypothetical zinc exporters identified, did not increase susceptibility to any of the divalent cations analyzed in this study, even though their encoded proteins display the canonical characteristics of known families of metal transporters. Thus, BOV_0501 shows characteristics of membrane proteins of the CDF family that transport metal divalent cations against a proton gradient (31) and that in prokaryotes usually function as dimers and contain approximately 300 amino acids, 6 TM domains, and a His-rich region—frequently located in the cytoplasmic CTD—that is able to bind metal cations and contribute to dimer stability (Figure 1A) (31–33, 54). The two His residues in the short cytoplasmic NTD (Figure 1A) might also be relevant for exporter activity, as reported for some representative members of bacterial CDF proteins (55). Additionally, BOV_0501 contains TM motifs associated with Zn^2+^ selectivity of the transporter: the L(X)_9_E motif in TM3 (31) and the H(X)_3_D and E(X)_3_D motifs located in TM2 and TM5 (31, 32, 55), respectively (Figure 1A). As observed for ZntA, BOV_A1100 displays the characteristic motifs of the actuator and ATP-binding domains of P_1B_-type ATPases (Figure 1B). However, motifs of its TM domain correspond to the P_1B-4_ subgroup that is mainly constituted by Co^2+^ exporters (30), although some of them also exhibit Zn^2+^ and Cd^2+^ efflux activity (56) that have only 6–7 TM regions and lack a cytoplasmic NTD (30, 43, 56). The activity of P_1B-4_-ATPases is highly dependent on four amino acid residues: a Met in TM1, the Ser and Cys residues of the TM4 SPC motif, and the His residue of the TM6 HEGGT(X)_5_N motif (43, 56) (Figure 1B). The three latter elements are found in BOV_A1100, but the Met residue is not present in TM1 (Figure 1B), which could explain, at least in part, why the Δ*BOV_A1100* mutant of *B. ovis* PA behaved as the parental strain in the presence of toxic levels of all the divalent cations tested (data not shown). However, two other factors could explain why a role as metal exporter was not detected for BOV_0501 and BOV_A1100: (i) the low level of transcripts of both genes observed in all conditions tested (data not shown) and (ii) the activity of *B. ovis* PA ZntA as exporter of divalent cations (Figures 4–6) that could compensate the hypothetical defects in metal efflux caused by the absence of BOV_0501 and BOV_A1100. Although overexpression of *BOV_0501* and *BOV_A1100* in the *B. ovis* PA Δ*zntA* mutant suggests that BOV_0501 and BOV_A1100 might contribute to *B. ovis* Zn^2+^ and Co^2+^ homeostasis, respectively (Figure 7), the low level of their transcripts in the conditions tested in this study, together with the absence of noticeable defects caused by their deletion, suggest a minor role for both genes in metal homeostasis.

Although none of the characterized mutants of rough *B. ovis* showed potential use as a vaccine candidate, the results presented in this study broaden knowledge about metal detoxification in pathogenic Brucellae that has only been studied in smooth species. The differences observed between *B. ovis* and *B. abortus* in the ZntR-ZntA system reveal a new source of heterogeneity among pathogenic *Brucella* species that, despite their high degree of homology at the DNA level, exhibit relevant differences in pathogenicity and host preference (57). The differences described here could also be related to the distinctive traits of *B. ovis* and *B. abortus* regarding outer membrane composition and properties (58, 59), which could also affect metal import–export pathways. Studies evaluating zinc import in *B. ovis* are being conducted in our laboratory to establish a more precise picture of zinc homeostasis in this rough *Brucella* species.

## Data availability statement

The raw data supporting the conclusions of this article will be made available by the authors, without undue reservation.

## Ethics statement

The animal study was reviewed and approved by the Bioethics Committee of the University of Salamanca and the competent authority of Junta de Castilla y León, Spain. The study was conducted in accordance with the local legislation and institutional requirements.

## Author contributions

BT-C: Conceptualization, Data curation, Investigation, Methodology, Writing – original draft, Writing – review & editing. CT: Data curation, Investigation, Methodology, Writing – review & editing. RC-Á: Investigation, Methodology, Writing – review & editing. PM: Investigation, Methodology, Writing – review & editing. NV: Conceptualization, Data curation, Formal analysis, Funding acquisition, Investigation, Methodology, Project administration, Supervision, Writing – original draft, Writing – review & editing.

## Footnotes

^1^ https://www.kegg.jp/kegg-bin/show_organism?org=T00534

^2^ https://www.kegg.jp/kegg/genes.html

^3^ https://blast.ncbi.nlm.nih.gov

^4^ https://www.bv-brc.org

^5^ https://wwwdev.ebi.ac.uk/Tools/jdispatcher/psa/emboss_needle

^6^ https://www.uniprot.org

^7^ https://phobius.sbc.su.se

^8^ https://www.psort.org/psortb/
