# Supplementary material for: Characterization of three predicted zinc exporters in Brucella ovis identifies ZntR-ZntA as a powerful zinc and cadmium efflux system not required for virulence and unveils pathogenic Brucellae heterogeneity in zinc homeostasis
Source: Front Vet Sci. 2024 Jan 8;10:1323500. doi: 10.3389/fvets.2023.1323500 (PMC10800456; doi:10.3389/fvets.2023.1323500)
Supplement: Supplementary file 1 [file Data_Sheet_1.PDF]

(A)

```
BOV_0501     1 ATGGGCGCCGATCACGAACATGCCGATGTGAAGAACACGCCGATTTCCCG  50
               ||||||||||||.||||||||                 ||||||||||||
BAB1_0523    1 ATGGGCGCCGATTACGAACAT-----------------GCCGATTTCCCG  33

BOV_0501    51 ATTATGGATCGCTTTCGGCCTGACCGGCATCTTCATGATTGCCGAAGTCA 100
               ||||||||||||||||||||||||||||||||||||||||||||||||||
BAB1_0523   34 ATTATGGATCGCTTTCGGCCTGACCGGCATCTTCATGATTGCCGAAGTCA  83
```

(B)

```
BOV_A1100    1 ATGGTTATAATATATGAAGTTTCCCAGGTCAGCCAGGCAAGCTTCATGCC  50
               ||||||||||||||||||||||||||||||||.|||||||||||||||||
BAB2_1160    1 ATGGTTATAATATATGAAGTTTCCCAGGTCAGTCAGGCAAGCTTCATGCC  50

BOV_A1100   51 CATATCCCTCACATCCGATCGTATGGCGATGGTACTCACCGGATTAACGA 100
               |||||||||||||||||.||||||||||||||||||||||||||||||||
BAB2_1160   51 CATATCCCTCACATCCGGTCGTATGGCGATGGTACTCACCGGATTAACGA 100

BOV_A1100  101 TCCTCGGTATCATTCCGGCCACATATGCCTTTTTTGCAGGTCCAAATGCC 150
               |||||||||||||||||||.||||||||||||||||||||||||||||||
BAB2_1160  101 TCCTCGGTATCATTCCGGCGACATATGCCTTTTTTGCAGGTCCAAATGCC 150

                 ↓              ↓                ↓               ↓

BOV_A1100  801 CGTTTCTTTTTACTGGCTTGGCTGGCCTGCGCGCGATGCGTTCTATCGGG 850
               |||||||||||||||||||||||.||||||||||||||||||||||||||
BAB2_1160  801 CGTTTCTTTTTACTGGCTTGGCTAGCCTGCGCGCGATGCGTTCTATCGGG 850
```

Supplementary Figure 1. Partial alignments of *BOV_0501* with its *B. abortus* 2308 ortholog (*BAB1_0523*) **(A)**, and of *BOV_A1100* with its *B. abortus* 2308 ortholog (*BAB2_1160*) **(B)**. Only the 5'-end of each gene that includes the *B. abortus* 2308 mutations is shown. The nt sequences of both genes in *B. ovis* PA are identical to those of the *B. ovis* 63/290 reference strain. In panel A, the 6-bp direct repeats present in *BOV_0501* at both sides of the *B. abortus* 2308 frameshift deletion of 17-bp, and that are probably involved in the deletion process by a slipped mispairing mechanism, are highlighted in bold red characters. The repeat conserved in *B. abortus* 2308 *BAB1_0523* is shown in bold and a potential ATG start codon for *BAB1_0523* is shown in blue. In panel B, the TGA translation stop codon generated by the nt substitution present in the *BOV_A1100* ortholog of *B. abortus* 2308 (*BAB2_1160*) is shown in bold red characters.
