# Supplementary material for: Characterization of three predicted zinc exporters in Brucella ovis identifies ZntR-ZntA as a powerful zinc and cadmium efflux system not required for virulence and unveils pathogenic Brucellae heterogeneity in zinc homeostasis
Source: Front Vet Sci. 2024 Jan 8;10:1323500. doi: 10.3389/fvets.2023.1323500 (PMC10800456; doi:10.3389/fvets.2023.1323500)
Supplement: Supplementary file 2 [file Data_Sheet_2.PDF]

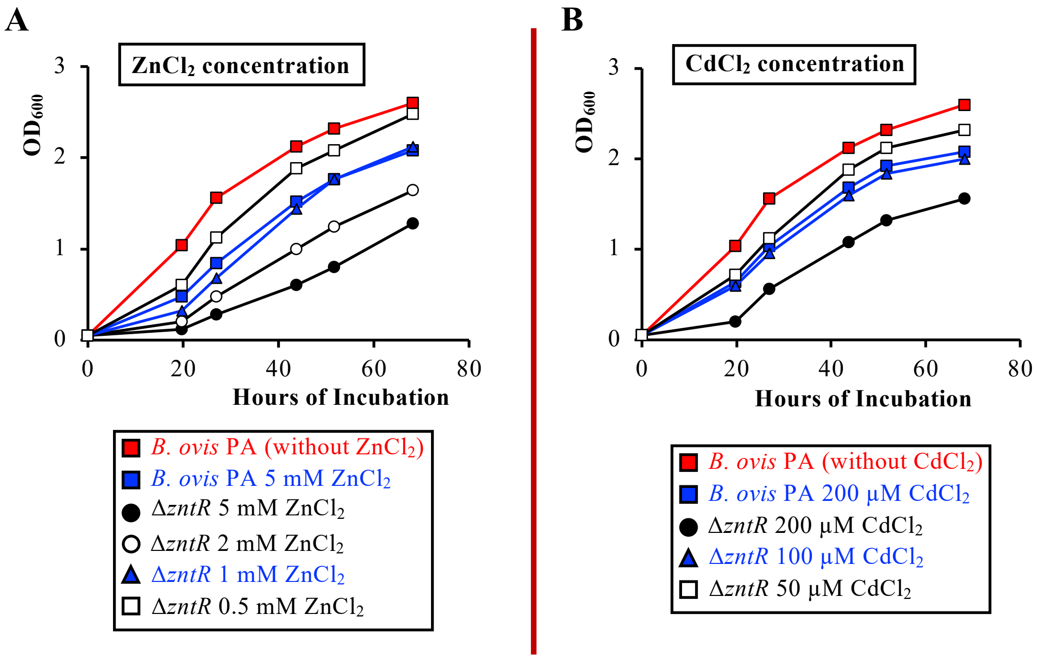

Supplementary Figure 2. Growth curves of the Δ*zntR* mutant or *B. ovis* PA in the presence of several $ZnCl_2$ concentrations **(A)**, and $CdCl_2$ concentrations **(B)**. Blue curves correspond to $ZnCl_2$ or $CdCl_2$ concentrations giving an equivalent growth pattern in parental *B. ovis* PA and its isogenic Δ*zntR* mutant.
